# Glia and Neural Stem and Progenitor Cells of the Healthy and Ischemic Brain: The Workplace for the Wnt Signaling Pathway

**DOI:** 10.3390/genes11070804

**Published:** 2020-07-16

**Authors:** Tomas Knotek, Lucie Janeckova, Jan Kriska, Vladimir Korinek, Miroslava Anderova

**Affiliations:** 1 Institute of Experimental Medicine, Czech Academy of Sciences, 1083 Videnska, 142 20 Prague, Czech Republic; tomas.knotek@iem.cas.cz (T.K.); jan.kriska@iem.cas.cz (J.K.); 2 Second Faculty of Medicine, Charles University, 84 V Uvalu, 150 06 Prague, Czech Republic; 3 Institute of Molecular Genetics, Czech Academy of Sciences, 1083 Videnska, 142 20 Prague, Czech Republic; lucie.janeckova@img.cas.cz (L.J.); vladimir.korinek@img.cas.cz (V.K.)

**Keywords:** Wnt signaling, central nervous system, neural stem/progenitor cell, glia, subventricular zone, subgranular zone, ischemia, stroke, adult neurogenesis

## Abstract

Wnt signaling plays an important role in the self-renewal, fate-commitment and survival of the neural stem/progenitor cells (NS/PCs) of the adult central nervous system (CNS). Ischemic stroke impairs the proper functioning of the CNS and, therefore, active Wnt signaling may prevent, ameliorate, or even reverse the negative effects of ischemic brain injury. In this review, we provide the current knowledge of Wnt signaling in the adult CNS, its status in diverse cell types, and the Wnt pathway’s impact on the properties of NS/PCs and glial cells in the context of ischemic injury. Finally, we summarize promising strategies that might be considered for stroke therapy, and we outline possible future directions of the field.

## 1. Introduction

In the central nervous system (CNS), Wnt signaling plays a pivotal role in embryogenesis, but it is also decisive postnatally, especially in regulating the proliferation, differentiation and migration of adult neural stem/progenitor cells (NS/PCs). In recent years, new links between Wnt signaling and a multitude of neuronal impairments affecting the CNS have emerged. Among others, schizophrenia [1], Alzheimer’s [2] and Parkinson’s diseases [3] are mental and neurodegenerative disorders that subvert the adult and aged mammalian brain. Nevertheless, this review mainly focuses on cerebral ischemia—acute brain injury associated with neurodegeneration. This medical condition negatively affects a broad spectrum of cells in the CNS, changing their behavior, functions and gene expression (reviewed in Reference [4]). However, there is a wide range of factors that might potentially help overcome the negative effects of ischemia. One of the cellular signaling pathways influenced by, and yet affecting the output of, ischemia is Wnt signaling. It has an indisputable function in the neuroprotection and regeneration of the CNS [5,6]. Wnt signaling affects the properties of NS/PCs and neuron-glial antigen 2 (NG2)-expressing cells, a glial type of cells that act as oligodendrocyte precursor cells (OPCs) in the healthy brain, and together with NS/PCs, they have a multipotent differentiation potential following brain injury [7,8,9]. This review summarizes recent findings on the role of the Wnt signaling pathway in the proliferation and differentiation of NS/PCs, and in the properties of other cell types in the CNS under ischemic conditions. Furthermore, we provide evidence that Wnt signaling should be considered in the treatment of ischemic brain injury.

## 2. Wnt Signaling

Wnt proteins act as important morphogens during embryogenesis, and their impact was also proven in postnatal ontogenetic stages, where they influence stem cell survival, proliferation, differentiation, and cell migration and polarity [10]. Before now, 19 distinct mammalian Wnt proteins have been identified [11]. They belong to a large group of secreted cysteine-rich proteins that are post-translationally modified by glycosylation and acylation. These modifications are required for the secretion of Wnts and for their full biological activity, respectively [12]. Glycosylated and palmitoylated Wnt molecules are subsequently bound to the Wntless (WLS) proteins, guided to the cell membrane of the Wnt-secreting cell, and released to the extracellular space [13]. The Wnt proteins activate different branches of Wnt signaling, three of which have been best characterized: the canonical—Wnt/β-catenin—pathway, and two non-canonical pathways—the planar cell polarity (PCP) pathway and the Wnt/calcium (Ca^2+^) pathway [14,15]. Various Wnt proteins, such as Wnt1, Wnt2b or Wnt3, initiate the canonical Wnt signaling branch, while Wnt5a and Wnt11 typically activate non-canonical Wnt signaling [16]. However, some Wnt proteins may play a dual role and activate both the canonical and non-canonical cascades simultaneously, in a tissue-specific manner [17,18]. In addition, non-canonical Wnts are able to antagonize the Wnt/β-catenin functions, and their balance thus allows the regulation of both the proliferation and differentiation of progenitor cells [19].

The canonical Wnt pathway regulates the stability of its key mediator β-catenin, which has a dual role in the cell. In epithelial cells, it plays a structural role in cell–cell adhesion. As a component of a protein complex with cadherins, the protein forms adherent junctions [20], while in a complex with α-catenin, it also interacts with the cytoskeleton [21]. On the other hand, free cytoplasmic β-catenin acts as an intracellular signal transducer in order to influence gene transcription [22], and exerts its full influence on the self-renewal and proliferation of NS/PCs [23]. In the absence of a Wnt signal, a multi-component, so-called β-catenin destruction complex is formed in the cytoplasm. It is composed of scaffolding proteins adenomatous polyposis coli (APC) and axis inhibition (AXIN), together with protein kinases glycogen synthase kinase 3β (GSK3β) and casein kinase 1 (CK1). Once β-catenin meets the destruction complex, it is phosphorylated at the N-terminal sequence by the kinases. Phosphorylated β-catenin is subsequently ubiquitinated by ubiquitin E3 β-transducin repeats-containing protein (β-TrCP), and eventually degraded in the proteasome. This leads to low intracellular levels of the protein, and leaves the nuclear transcription factors of the pathway T-cell factor/lymphoid enhancer-binding factor (TCF/LEF) silenced by co-repressors groucho and transducin-like enhancer of split (TLE) [24]. Nevertheless, once a Wnt protein exerts its ligand function and attaches to the frizzled (FZD) receptor and its co-receptor low-density lipoprotein receptor-related protein 5/6 (LRP5/6), the FZD-LRP receptor complex binds the dishevelled (DVL) protein. This protein, together with LRP phosphorylated at its intracellular domain, recruits AXIN to the plasma membrane [25]. These events lead to the disassembly of the destruction complex and, as a result, stabilized β-catenin accumulates in the cytoplasm, and is thereafter translocated to the nucleus. Moreover, another mechanism bypassing the AXIN protein has been recognized, where LRP6 directly inhibits GSK3β and thus stabilizes β-catenin independent of the destruction complex [26]. In the nucleus, β-catenin binds to the effector transcription factors from the TCF/LEF family, subsequently binding other co-activators in order to initiate transcription of the Wnt-responsive genes. Some of the genes influence the progression of the cell cycle (e.g., cyclin D1) and are characteristic of adult stem cells (e.g., leucine-rich repeat-containing G protein-coupled receptor 5, LGR5) [27], while other Wnt target genes negatively regulate the pathway (e.g., AXIN) [28]. For further information on canonical Wnt signaling, please visit the Wnt homepage [29].

Unlike the canonical Wnt pathway, the non-canonical PCP pathway orchestrates the orientation of the cells that reside in the tissue [30,31]. At the outset of signaling, Wnt ligands bind to the FZD receptor and receptor tyrosine kinases of the RYK and ROR families [32] to subsequently transduce the signal to DVL. The relay does not involve β-catenin, but instead is mediated by the small GTPase Ras homolog family member A (RhoA) and the Rac family small GTPase 1 (RAC1). RhoA activation also requires the DVL-associated activator of morphogenesis 1 (DAAM1) protein, and via Rho-associated kinase (ROCK), it mediates actin polymerization. RAC1, together with another GTPase, CDC42, further activates c-Jun N-terminal kinase (JNK), which is also implicated in the rearrangement of the cytoskeleton. Moreover, JNK phosphorylates transcription factor C-JUN, which forms a heterodimer with the activator protein 1 (AP1) transcription factor and thus controls cell proliferation, differentiation and apoptosis [33].

**Figure 1:**
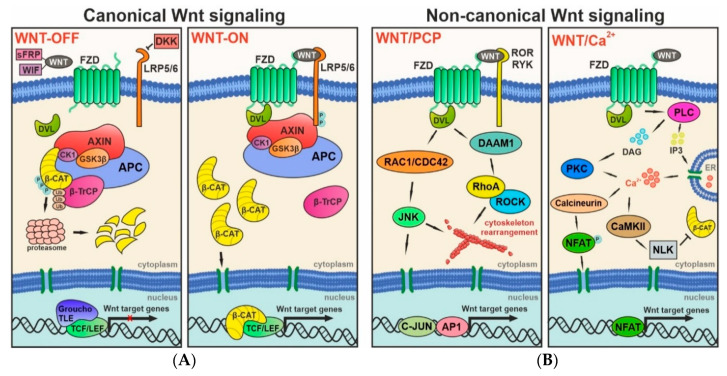
Overview of the canonical and non-canonical Wnt signaling pathways. (**A**) β-catenin-dependent signaling is activated by binding of a canonical Wnt ligand to the frizzled (FZD) receptor and the low-density lipoprotein receptor-related protein 5/6 (LRP5/6) co-receptor. Subsequently, β-catenin is released from the destruction complex and translocated to the nucleus, where it forms a complex with the T-cell factor/lymphoid enhancer-binding factor (TCF/LEF) proteins in order to activate transcription of the Wnt-responsive genes; (**B**) Activation of the non-canonical—β-catenin-independent—Wnt signaling branches influences cell properties and results in transcription of alternative Wnt target genes. In addition, in some cells non-canonical signaling inhibits β-catenin-mediated transcription. A more detailed description of the pathways is given in the text. Abbreviations: AP1, activator protein 1; APC, adenomatous polyposis coli; AXIN, axis inhibition; β-CAT, β-catenin; β-TrCP, β-transducin repeats-containing protein; C-JUN, transcription factor C-JUN; Ca^2+^, calcium; CaMKII, Ca^2+^/calmodulin-dependent protein kinase II; CDC42, GTPase CDC42; CK1, casein kinase 1; DAAM1, DVL-associated activator of morphogenesis 1; DAG, diacylglycerol; DKK, dickkopf; DVL, dishevelled; ER, endoplasmic reticulum; GSK3β, glycogen synthase kinase 3β; IP3, inositol trisphosphate; JNK, c-Jun N-terminal kinase; NFAT, nuclear factor of activated T-cells; NLK, nemo-like kinase; P, phosphorylation; PCP, planar cell polarity; PKC, protein kinase C; PLC, phospholipase C; RAC1, Rac family small GTPase 1; RhoA, Ras homolog family member A; ROCK, Rho-associated kinase; ROR, receptor tyrosine kinase ROR; RYK, receptor tyrosine kinase RYK; sFRP, secreted FZD-related proteins; TLE, transducin-like enhancer of split; Ub, ubiquitination; WIF, Wnt inhibitory factor.

The Wnt/Ca^2+^ pathway primarily regulates cell adhesion and motility. Upon Wnt signaling activation, the signal is relayed through phospholipase C (PLC), which hydrolyzes phosphatidylinositol 4,5-bisphosphate (PIP2) to inositol trisphosphate (IP3) and diacylglycerol (DAG). IP3 triggers Ca^2+^ release from the endoplasmic reticulum. Elevated intracellular Ca^2+^ concentration together with DAG activate Ca^2+^-dependent enzymes, such as protein kinase C (PKC), calcineurin, and Ca^2+^/calmodulin-dependent protein kinase II (CaMKII). While PKC downstream signaling promotes cytoskeleton remodeling in the PCP pathway [34], calcineurin activates the nuclear factor of activated T-cells (NFAT) transcription factor, which further regulates cell proliferation and differentiation. The CaMKII-dependent branch of the pathway activates the nemo-like kinase (NLK), which antagonizes the Wnt/β-catenin pathway [35]. The main components of the three Wnt branches are outlined in Figure 1.

Additionally, Wnt signaling may be suppressed or regulated by a plethora of extracellular proteins. Secreted FZD-related proteins (sFRPs) and Wnt inhibitory factor (WIF) both bind to Wnt proteins, and thus chelate the ligands that may potentially activate Wnt signaling. Therefore, they act as antagonists for both the canonical and non-canonical pathways [36]. Another class of Wnt inhibitors belongs to the dickkopf (DKK) family. These proteins have been shown to block Wnt signaling by disrupting the FZD-LRP receptor complex [37], thus specifically antagonizing the Wnt/β-catenin pathway [38]. All the aforementioned extracellular molecules affect Wnt signaling in a short-range manner and inhibit only the cells residing in the close vicinity of the ligand-secreting cell [39]. Moreover, the interpretation of the differentially expressed components of signaling pathways may be challenging, as many of them do not act separately, and rather share multiple pathways. In addition, Wnt signaling also interacts with other pathways that are involved in NS/PC fate specification and adult tissue homeostasis, such as sonic hedgehog (Shh) signaling. Wnt and Shh both influence NS/PC maintenance, proliferation and differentiation [40,41]. It has been shown that the coordination of Wnt and Shh signaling via the glioma-associated oncogene 3 (GLI3) protein alters the type of NS/PCs-derived neurons [42]. Additionally, GLI1 and GLI2 both inhibit Wnt signaling through positive regulation of the Wnt blocker sFRP1, whereas, concurrently, β-catenin and TCF/LEF can enhance the transcriptional activity of the GLI1 protein and thus promote Shh signaling [43].

## 3. Wnt Signaling in Adult Neurogenesis of the Healthy Brain

The Wnt signaling pathway in the adult brain, as in many other tissues, is indispensable in the regions where new cells are generated. In the healthy CNS, Wnt signaling plays multiple roles in the self-renewal, neurogenesis and homeostasis of neural stem cells (NSCs). Adult NSCs undergo asymmetric division to maintain a pool of pluripotent cells, while providing progenitor cells capable of differentiating to neuronal and glial cell populations. Recent studies provide evidence that these processes require precise regulation of molecular signals in order to prevent the gradual loss of postnatal NSCs, and ensure continuous neurogenesis in the brain (reviewed in Reference [23]).

In the mammalian brain, adult neurogenesis is limited to two distinct regions where doublecortin (DCX)-positive putative neuroblasts occur—the dentate gyrus (DG) of the hippocampus (subgranular zone, SGZ) [44] and the vicinity of the walls of the lateral ventricles (LVs; subventricular zone, SVZ) [45]. In comparison to embryonic development, the number of postnatal neuroblasts in these areas is very low, and further declines with age. Moreover, the controversial results of recent studies have given rise to doubts regarding whether substantial neurogenesis actually occurs in the SGZ of the adult human brain [46,47]. In any case, the possibility of deciphering how the neuroblasts in this region actually contribute to the pool of mature neurons, and whether they are involved in the brain activity for a long period of time, is limited within human tissue. Studies in experimental animals, especially mice, provide a more detailed look into adult neurogenesis in both areas. In brief, adult NSCs are mainly quiescent and only a small portion of them is activated, creating an equilibrium state that protects the tissue from exhaustion of the NSC pool. Activated NSCs give rise to proliferating neural progenitor cells (NPCs), differentiating to neuroblasts, which gradually mature to terminally differentiated neurons. However, many studies show the existence of different types of NSCs that have the potential to differentiate to distinct types of neurons and, in addition, to several types of glial cells. Such NSCs differ in localization within the SVZ and the SGZ, as well as in the expression of some transcription factors upon their activation [48,49]. However, they share many common markers, such as glial fibrillary acidic protein (GFAP), nestin, and SRY-box transcription factor 2 (Sox2), and they are in contact with blood vessels (reviewed in Reference [50]).

Adult neurogenesis is a process including the proliferation and specification of neural progenitors, their differentiation to neurons, and neuron migration and integration into the existing circuits. The active canonical Wnt pathway, together with the removal of Sox2-mediated repression, leads to the activation of quiescent NSCs and drives their proliferation, most likely through the expression of the gene encoding transcription factor neurogenic differentiation 1 (NeuroD1) and through the activation of long interspersed nuclear elements 1 (LINE1) [51]. Wnt signal is produced by adult hippocampal progenitors and astrocytes near the SGZ [52,53], and adult Wnt7 knock-out mice display decreased proliferation and, moreover, decreased Dcx-expressing neuroblasts in both the hippocampus and the SVZ [54]. In the SVZ, retrovirus-mediated expression of a stabilized form of β-catenin was shown to induce proliferation of NPCs [55]. Conversely, the loss of *disrupted in schizophrenia 1* gene (Disc1) has been shown to reduce the proliferation of adult NPCs in the hippocampus through Wnt/β-catenin inhibition [56]. Besides NS/PC maintenance and proliferation, Wnt7 has been shown to promote neuronal differentiation and maturation via β-catenin/neurogenin 2 signaling in the DG [57]. In addition, β-catenin-Tcf/Lef signaling enhanced neuronal differentiation in this region through the expression of the prospero-related homeodomain transcription factor 1 (Prox1) [58]. Moreover, enhanced β-catenin/homeodomain interacting protein kinase 1 (Hipk1) resulted in decreased proliferation and increased differentiation of NS/PCs in the SVZ [59], indicating that the precise balancing of the Wnt/β-catenin signaling level is indispensable in maintaining long-term neurogenesis in the adult brain. In addition, the gradual loss of neurogenesis related to aging is associated with the attenuation of the canonical Wnt signaling pathway, as illustrated by the decreased expression of Wnt3 ligands in the DG in aged mice [60]. The age-related decrease of neurogenesis has also been linked to the expression of Wnt inhibitory molecules in both the hippocampus, where Dkk1 [61] and sFRP3 [62] were expressed, and the SVZ, where the expression of wild-type p53-induced phosphatase 1 (Wip1) and Dkk3 was observed [63]. Strikingly, the loss of Dkk1 expression restored neurogenesis and improved the cognitive functions of aged mice [64]. On the other hand, induced Dkk1 expression in the adult hippocampus resulted in the loss of neuronal synapses and long-term memory deficits [65]. Notably, the production of Wnt inhibitory molecules in healthy adult tissue is likely to be regulated by multiple pathways whose potency decreases with age, as shown on the p38 mitogen-activated protein kinase (MAPK) pathway in the adult SVZ [66].

The non-canonical Wnt signaling pathway also plays a role in adult neurogenesis, as evidenced by behavioral changes and reduced long-term memory in mice deficient of JNKs [67,68]. Constitutive expression of non-canonical ligand Wnt5a was documented in the adult hippocampus. Through the JNK pathway, non-canonical Wnt plays a role in neuronal differentiation and synapse modulation [69,70]. FZD5/JNK signaling is involved in the formation of the neuronal polarity and development of neuronal processes [71]. A role of the downstream c-Jun effector AP1 in neuronal maturation was shown previously [72]; moreover, Wnt/JNK/AP1 signaling promoted differentiation to neurons from induced pluripotent stem cells (iPSCs) in vitro in a β-catenin-independent manner [73]. A more recent study then identified the Wnt/JNK and Wnt/CaMKII signaling pathways (activated by the Wnt5a ligand) as essential players in the neuronal differentiation of NPCs in the adult hippocampus [74]. In contrast, both canonical and non-canonical Wnt ligands—Wnt3a and Wnt5a—increased the level of β-catenin and, subsequently, NPC proliferation and differentiation in SVZ-derived cells [75]. Moreover, adaptor protein ATPase H^+^ transporting accessory protein 2 (ATP6AP2) was identified as a regulator of the cell response from Wnt/β-catenin to Wnt/PCP signaling in adult NSCs isolated from the hippocampus [76]. In addition, the attenuation of non-canonical Wnt signaling promoted the activation of quiescent NCSs in the adult SVZ [77]. All of these observations suggest that both the canonical and non-canonical Wnt pathways are required for the production of mature neurons, and the mutual balancing of these often opposing pathways is necessary to maintain neurogenesis in the adult brain.

## 4. Wnt Ligand Expression/Sensing in Particular Cell Types of the Adult Neurogenic Regions

**Figure 2:**
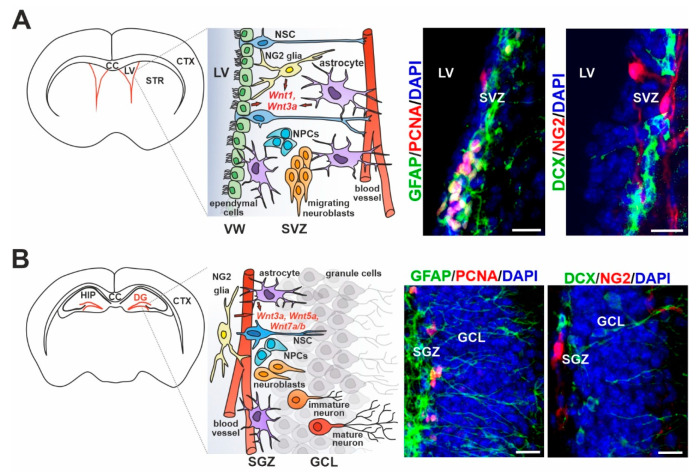
Neurogenic niches of the adult mammalian brain. Schematic diagrams illustrating the main cellular components within the neurogenic niche in the (**A**) subventricular zone (SVZ) of the lateral ventricles (LVs) and (**B**) dentate gyrus (DG) of the hippocampus (HIP) containing the neurogenic niche in the subgranular zone (SGZ), and representative immunofluorescence images of the corresponding regions showing the main markers of cell types indicated in the illustrated schemes. The neuronal lineage in both compartments is represented by neural stem cells (NSCs), neural progenitor cells (NPCs) and migrating neuroblasts. In addition, neuronal maturation is shown in the granule cell layer (GCL) adjacent to the SGZ. Glial cells, represented by astrocytes and neuron-glial antigen 2 (NG2)-positive glia in both regions, and by ependymal cells constituting the ventricular wall (VW) of the LVs, release Wnt ligands (in red color) in order to activate quiescent NSCs and mediate their proliferation/differentiation. Glial fibrillary acidic protein (GFAP) represents NSCs and a subpopulation of astrocytes; proliferating cell nuclear antigen (PCNA) marks proliferating progenitors; doublecortin (DCX)-positive cells identify both neuroblasts and immature neurons; NG2 is present in NG2 glia; 4′,6-diamidino-2-phenylindole (DAPI) stains cell nuclei. Note that the magnified field and DG histology in panel B are rotated approximately 90° counterclockwise compared to the scheme of the frontal section. Scale bar: 15 µm. Abbreviations: CC, corpus callosum; CTX, cortex; STR, striatum.

Considering the activity of the Wnt signaling pathways in the neurogenic regions of the adult brain, it is necessary to distinguish the Wnt-signal-producing cell types from the Wnt-receiving cells in the region. As in other adult tissues with homeostatic capacity (e.g., the intestine, stomach or skin; reviewed in Reference [78]), NSCs in the adult brain need their niche that produces the signals essential for NSC maintenance and proliferation (Figure 2). The adult neurogenic niche includes glial cells, specifically astrocytes and NG2 glia, and also, in the case of the SVZ, ependymal cells. Glial cells represent a large proportion of cells, between one and two thirds of all cells in the adult mammalian brain; in humans, the number of neurons and glial cells is almost equal [79]. Although glia were previously mainly considered as supportive cells, studies over the last 20 years have clearly shown their indispensable role in adult neurogenesis under physiological conditions and in brain tissue regeneration after injury.

Astrocytes are present in all parts of the brain and provide many supporting functions, such as the maintenance of tissue homeostasis and the blood–brain barrier (BBB), but they also participate in the tripartite synapses (reviewed in Reference [80]). A subset of GFAP-producing astrocytes is crucial for neuronal health and integrity, as their removal leads to severe neuronal degeneration [81,82]. Astrocytes in the adult neurogenic niches serve as a source of GFAP-positive NSCs, and share the functional characteristics of glial cells and multipotent progenitors [83]. In the SGZ, astrocytes promote NSC proliferation and neuronal differentiation [52] by producing the canonical Wnt3a ligand in cooperation with bone morphogenetic proteins 2 and 4 (BMP2/4) [84]. In the SVZ, astrocytes were shown to produce Wnt7a, which stimulates the canonical branch of the pathway [85]. Astrocytes outside the neurogenic niche in the healthy adult brain appear to fulfil different functions, and show neither progenitor properties nor neurogenic potential. However, their properties change after traumatic brain injury and stroke (reviewed in Reference [86]).

NG2-expressing glial cells, also called polydendrocytes, are the most recently discovered type of glial cells [87]. NG2 glia are highly proliferative and harbor several stem cell characteristics. As precursor cells, they mainly generate myelinating oligodendrocytes in cell culture and in the healthy adult brain, and are therefore often called OPCs. Nevertheless, it has been discovered that NG2 glia form synapses with neurons and are able to receive their signal, although they themselves are not capable of generating action potentials and transmitting signals to other cells (reviewed in Reference [80]). Importantly, these cells have a high repopulation capacity [88,89] that declines with age [90,91]. The chronic loss of this cell type in the white matter of the cerebral cortex resulted in reduced oligodendrogenesis, which subsequently led to motor dysfunctions [92]. Interestingly, their ablation in the prefrontal cortex caused deficits in excitatory glutamatergic neurotransmission and astrocytic glutamate uptake, and led to depressive-like behaviors in mice [93], indicating more than one role for NG2 glia in the adult brain. Furthermore, in vitro studies revealed the multipotent capacity of these cells, as they were able to differentiate to functional neurons or astrocytes [94,95]. Multiple follow-up studies using genetic fate mapping have excluded the differentiation of NG2 glia to cell types other than oligodendrocytes (and NG2 glia in order to repopulate their pool) in the adult brain [96,97,98], but recent research has indicated that this potential may be activated in the event of traumatic or ischemic injury [99,100,101,102].

The expression of the platelet-derived growth factor receptor α (PDGFRα) is essential for the differentiation of NG2 glia to oligodendrocytes [103], and through the activation of the Wnt/β-catenin pathway it also potentiates the proliferation of polydendrocytes [104,105]. Surprisingly, several studies have demonstrated that Wnt/β-catenin signaling negatively regulates oligodendrogenesis during embryonic development [106,107] and, in cooperation with the BMP signaling pathway, delays oligodendrocyte maturation in vitro in postnatal rodent NG2 glia [108,109]. On the other hand, Wnt-negative regulator APC has been reported to be transiently enhanced in newly differentiated oligodendrocytes and, moreover, it co-localized with Wnt effector TCF4 in the same cell population, regulating oligodendrogenesis in both a β-catenin-dependent and a β-catenin-independent manner [110]. Furthermore, the recombinant canonical Wnt3a ligand potentiated differentiation of adult NG2 glia expressing *Olig2* in cell cultures isolated from the adult SVZ without affecting the neurogenesis from these cells [111]. Consistently, GSK3β inhibition (resulting in Wnt/β-catenin signaling activation) stimulated the expression of myelin basic protein (MBP) and oligodendrocyte maturation in vitro and in vivo [112,113]. In addition, fluorescence-activated cell sorting (FACS) analysis revealed that Wnt3, the most abundant Wnt ligand in the adult SVZ, is predominantly expressed by NG2 glia, suggesting a positive feedback loop driving the generation of this cell type from adult SVZ NSCs [111].

NS/PCs in both neurogenic regions—the SVZ and the SGZ of adult mice—were shown to express the Wnt/β-catenin-responsive gene *Axin2* [114], and many other studies reported active Wnt/β-catenin signaling in adult NS/PCs present in these regions [55,56,58,59]. Transplantation experiments showed that extrinsic sources of the signal from the neurogenic niche are essential for the maintenance of NSCs, as well as for the neuronal fate and specification of neuronal subtypes [115,116,117]. In fact, adult NS/PCs represent a diverse pool of cells with high plasticity, and their surrounding niche is what determines their future characteristics. Consequently, adult tissue is able, although to a limited extent, to respond to its current needs, and is capable of at least partial regeneration after traumatic injury (reviewed in Reference [118]). In addition, many studies suggest that adult NSCs are not only the recipients of the Wnt signal, but they might also secrete Wnt molecules. The canonical Wnt ligands Wnt3 and Wnt7a were found in adult hippocampal NPCs [52,53], and high expressions of non-canonical Wnt ligand Wnt5a and canonical ligands Wnt7a and Wnt7b were found in vitro in NS/PCs isolated from the neonatal SVZ. Importantly, enhanced levels of Wnt/β-catenin signaling resulted in increased differentiation to neuronal precursors, and conversely, decreased canonical Wnt signaling potentiated gliogenesis [119]. This is consistent with a more recent study in which the Wnt/β-catenin pathway suppressed astrogliogenesis during spinal cord development in vivo [120]. On the other hand, transcriptional profiling did not reveal the expression of any Wnt ligands in NSCs isolated from the adult SVZ, and conversely, Dkk3 was highly produced in NSCs. This observation could be attributed to the predominant quiescent state of adult NSCs in the SVZ [121]. In the same study, Azim and colleagues found expressions of Wnt ligands in the surrounding neurogenic niche, where Wnt7b was localized in glial cells. In addition, the enrichment of mRNAs encoding several components of the Wnt pathway was recorded in endothelial cells found in the circumjacent tissue [121]. As shown recently, Wnt/β-catenin signaling in NSCs plays a role primarily in maintaining the BBB [122,123]. However, the role of Wnt signaling in adult neurogenesis is still enigmatic.

Ependymal cells are present only in the SVZ, constituting a multiciliated lining of the LVs. They form pinwheel-like structures in order to surround the apical processes of NSCs attached to the basal lamina of the LVs [124]. Their structural function seems to be essential for the maintenance and differentiation of NSCs and the migration of their progeny, as evidenced by reduced neurogenesis and impaired neuroblast migration upon the loss of ependymal cells in the SVZ [125]. The expression of Wnt ligands Wnt1, Wnt3, Wnt5a and Wnt11, and several Wnt signaling components, was observed in the ependymal layer of the neurogenic region of the spinal cord surrounding the central canal [126,127]. Moreover, experiments based on the detection of the Wnt-responsive gene *Axin2* revealed the active Wnt/β-catenin pathway in adult ependymal cells residing in the SVZ [114]. In addition, ependymal PCP induced by non-canonical Wnt/PCP signaling [128] is involved in neuronal migration, as motile cilia of ependymal cells generate cerebrospinal fluid (CSF) flow that guides newly generated neurons [129].

## 5. Cerebral Ischemia

Stroke is a major cause of death and disability worldwide, and based on its etiology, it can be either ischemic or hemorrhagic. Ischemic stroke is caused by a reduction in the cerebral blood flow, whereas hemorrhagic stroke is caused by intracerebral bleeding. In this review, we focus on ischemic stroke, since it is the predominant form, accounting for approximately 80% of all stroke cases [130]. Ischemia is characterized as a decrease in the blood flow, sufficient to affect normal cell functions. Brain tissue is extremely prone to ischemic injury due to its high energy demand, and even brief periods of ischemia can lead to serious damage and cell death [131].

Based on its extent and origin, ischemic brain injury may be subdivided into global cerebral ischemia (GCI) and focal cerebral ischemia (FCI). While FCI only affects a certain region of the brain, GCI is caused by decreased blood flow in the whole brain. Common causes of FCI are thrombosis and embolism, whereas GCI occurs after systemic hypoperfusion or asphyxia [131,132]. In the brain region affected by FCI, two zones are recognized. A zone of severe ischemic injury, the ischemic core, is characterized by low adenosine triphosphate (ATP) levels, high concentrations of glutamate and ions, and acidosis. Necrosis is the prevailing type of cellular death in this area. However, the severity of the damage decreases gradually with the distance from the core, in a region known as the ischemic penumbra. In this region, the residual blood flow from collateral arteries mitigates the conditions. A drop in ATP levels is milder, causing transient ischemic depolarization and ion shift in the penumbra, while glutamate concentration rises during the first hour after ischemia, but then declines to the physiological levels. The conditions during GCI are similar to those in the ischemic core [133].

The reduction in the blood flow leads to a deficit in glucose and oxygen in the brain tissue, thus damaging cellular energy metabolism. A quick depletion of ATP follows, and leads to the loss of the membrane potential and depolarization due to the failed maintenance of ionic gradients by ion pumps, followed by glutamate release from neurons. This ischemic pathway thus leads to the process known as glutamate excitotoxicity (reviewed in Reference [4]). The activation of glutamate receptors further increases intracellular concentrations of Ca^2+^, together with elevated intracellular concentrations of sodium (Na^+^) and chloride ions (Cl^−^), followed by a passive influx of water and edema formation [134]. Simultaneously, the high concentration of Ca^2+^ leads to the activation of Ca^2+^-dependent enzymes, such as phospholipase A_2_ and cyclooxygenase. This results in the production of reactive oxygen species (ROS) and the subsequent damage of genomic DNA, proteins, lipids and the cell membrane [135]. One of the main targets of ROS are mitochondria. Radicals can induce the disruption of the inner mitochondrial membrane and further negatively affect ATP production. Moreover, damaged mitochondria release pro-apoptotic signals, leading to cell death [136]. Ischemic damage and processes occurring thereafter lead to neuroinflammation, in which glial cells, particularly microglia, but also astrocytes, are involved [137]. Moreover, the BBB is damaged after ischemic injury, loses its function, and becomes permeable for fluids and plasma elements [138].

In reaction to FCI, a structure known as the glial scar is formed. Within hours after ischemic injury, macrophages and microglia appear, and subsequently, NG2 glia emerge in the tissue surrounding the injured region within a few days after the insult. A permanent glial scar consists predominantly of astrocytes, but it also contains microglia and NG2 cells [101,139]. The formation of the glial scar is a process that occurs both in experimental animal models of stroke, and in humans [140]. A more detailed description of ischemic stroke and glial scar formation is provided elsewhere [4,131].

## 6. The Impact of Wnt Signaling on Different Cell Types during Ischemia

Wnt signaling has been shown to play an important role after ischemic injury in the brain. The current treatment of ischemic stroke is limited to restoring the blood flow through the occluded artery [141]; however, Wnt signaling has been documented to affect various biological processes that might help with tissue recovery.

One such process might be the regeneration of brain vascularization. The activation of Wnt signaling by Wnt3a caused an increase in the BBB-like function of cultured human brain endothelial cells [123]. The Wnt7a and Wnt7b ligands, produced by neuroepithelium during the mouse brain development, were observed to target the vascular endothelium and stimulate formation of the BBB by activation of the canonical Wnt signaling pathway [142]. It is evident that this role of Wnt signaling is maintained in postnatal development [143]. Treatment of ischemic rats with compound porcine cerebroside and ganglioside injections led to the amelioration of the injury by promoting the cerebral blood flow, and simultaneously, an increased production of Wnt signaling pathway-associated proteins was noted. Taken together, the indicated results imply that Wnt signaling might play a role in the mitigation of impacts caused by cerebral ischemia [144].

Wnt signaling does not only impact the restoration of the blood flow after ischemic injury, but it has also been described as a neuroprotective agent. The concentration of the Wnt signaling pathway antagonist Dkk1 rose in the rat hippocampus after transient GCI, while restoration of the pathway protected hippocampal neurons against the damage caused by ischemia [145]. A similar phenomenon was observed in rats undergoing FCI. Moreover, neuronal damage caused by the middle cerebral artery occlusion (MCAO), a commonly utilized animal model of FCI, was reduced in mice with decreased expression of Dkk1 [146]. A possible explanation of this effect is that β-catenin regulates the expression of B-cell lymphoma 2 (Bcl2), a protein with anti-apoptotic properties [147]. Interestingly, post-conditioning with isoflurane reduced the infarction volume and apoptosis of neurons in rats, and this effect was linked to Wnt signaling activation [148]. Since elevated plasma levels of Dkk1 were observed in humans after stroke [149], we hypothesize that similar mechanisms might occur in the human brain.

Apart from its function in neuroprotection, Wnt signaling has been reported to play a role in nervous tissue regeneration after stroke. Many studies have reported that ischemic injury leads to increased neurogenesis in the SVZ and SGZ [150]. Since Wnt signaling increases neurogenesis in the healthy brain, it should be considered a possible contributor to ischemia-induced neurogenesis. Indeed, an increase in Wnt signaling, together with increased symmetrical division of NSCs, was observed after stroke in the SVZ, and blocking the Wnt pathway led to a drop in the number of neurospheres [151]. Moreover, Wnt3a administration led to a decreased infarction zone size. Furthermore, treatment with Wnt3a increased the expression of brain-derived neurotrophic factor (BDNF) and promoted the proliferation of neuroblasts in the SVZ. At 14 days after the onset of stroke, neuroblasts migrated to the peri-infarct region, and at 21 days after ischemic injury, increased numbers of newly generated neurons were observed [152]. In mice suffering from FCI in the striatum, the over-expression of Wnt3a led to increased proliferation of NSCs that later became mature neurons, improving motor functions 28 days after injury. Furthermore, Wnt3a over-expression in the SVZ caused an increase in the number of proliferating cells, accompanied by a reduction in size of the lesion two days after ischemia. Additionally, increased numbers of immature neurons in the striatum were observed [153]. Conversely, the inhibition of Wnt signaling by β-catenin-specific small interfering RNA (siRNA) after ischemic injury led to reduced numbers of proliferating progenitor cells, and both immature and mature neurons [154]. Interestingly, the level of β-catenin in the ischemic striatum was increased by the Bcl2 protein, and this increase was linked to enhanced neurogenesis. The neurogenic effect of Bcl2 was reversed by the administration of β-catenin siRNA, indicating the involvement of the Wnt signaling pathway [155]. A similar effect was observed in hemorrhagic stroke, where Wnt signaling activation promoted neurogenesis in the SVZ together with Bcl2 and β-catenin expression, suggesting that Wnt signaling plays similar roles in both ischemic and hemorrhagic stroke [156].

The neuroprotective and regeneration-stimulating effects of Wnt signaling after ischemic injury were also evidenced by the promotion of neurogenesis mediated by cadherin epidermal growth factor laminin G seven-pass G-type receptor 1 (CELSR1). The upregulation of *CELSR1* mRNA was observed in the rat SVZ after ischemic stroke, and it led to increased neurogenesis and angiogenesis, mainly via the Wnt/PKC pathway [157]. Neuroglobin, an oxygen-binding globin protein known for its neuroprotective properties, was shown to increase neurogenesis in the mouse brain affected by transient FCI. Again, this effect was described to act at least partially via canonical Wnt signaling [158].

An insight into a native mechanism by which the damaged tissue increases Wnt signaling was obtained in in vitro cultures of adult rat NSCs under hypoxic conditions. Under these circumstances, increased levels of β-catenin and increased neurogenesis were observed. Simultaneously, hypoxia-inducible factor 1α (HIF1α) expression was upregulated, and the inhibition of this factor counteracted hypoxia-induced expression of β-catenin [159]. A further insight into this mechanism was provided by Chen and colleagues, who reported that after ischemia-reperfusion injury in rats, peroxynitrite was produced, activating the proliferation and differentiation of NSCs via the upregulation of HIF1α and Wnt signaling. However, higher doses of peroxynitrite were neurotoxic [160]. Another intrinsic factor that might play a role in the reaction of nervous tissue to ischemic injury is microRNA (miRNA)-148b, since its over-expression has been reported in the SVZ of the ischemic rat brain. The inhibition of this particular miRNA enhanced the expression of Wnt1 and β-catenin. Moreover, miRNA-148b inhibition caused increased proliferation of NSCs and their differentiation to neurons and astrocytes [161], suggesting that the ischemia-induced (over)expression of miRNA-148b had detrimental effects on the brain tissue, and might be a possible therapeutic target. Wnt signaling was also affected in mice with knock-out of the *Ppm1d* gene encoding Wip1 phosphatase. Animals of this mouse strain exhibited worsened functional recovery after ischemia, which was compensated for by pharmacological activation of the Wnt signaling pathway. This provided further understanding of Wnt-related mechanisms occurring in the nervous tissue after stroke [162]. A different Wnt-related mechanism affecting neurons and NSCs in the ischemic brain was associated with myeloperoxidase-dependent oxidative stress mediating decreased β-catenin levels after ischemic injury. Inhibiting myeloperoxidase with N-acetyl lysyltyrosylcysteine amide counteracted Wnt signaling suppression, and led to increased numbers of NSCs and immature neurons in the cortex [163]. Additionally, long non-coding RNA maternally expressed gene 3 (Meg3) was found to act as a negative regulator of neurogenesis. Meg3 was increased after ischemia-reperfusion injury in rats, and reduced expression of this RNA molecule promoted neurogenesis, reduced the infarct area, and attenuated neuronal apoptosis and necrosis. Moreover, the effect of the decreased Meg3 levels was reversed by treatment with Dkk1, while the detrimental impact of Meg3 was reversed by lithium chloride (LiCl), a Wnt signaling activator [164]. Interestingly, NSCs were also affected by tissue plasminogen activator (tPA), a substance that is often used for ischemic stroke treatment to dissolve the thrombus. Inhibiting the tPA activity in primary cultures caused decreased neurite outgrowth—the same phenotype as observed in the knock-down of β-catenin or LRP5/6. Moreover, axon density was lower in the ischemic rat brain treated with transplanted NSCs with tPA knock-out, when compared to the brains treated with wild-type NSCs [165]. This suggests that tPA produced by NSCs might affect the ischemic tissue via the Wnt signaling pathway.

Taken together, there is strong evidence that Wnt signaling is crucial in stimulating NS/PC proliferation after ischemic injury. Strikingly, Morris and co-workers showed that the expression of Wnt genes was not upregulated in the SVZ of rats after ischemic injury [166]. Contrary to this observation, another study reported increased cell numbers with active Wnt signaling in the SVZ after stroke [151]. This discordance might be due to the different regions examined or the use of different methods. While Morris and colleagues used cultured NSCs from ischemic rats, and analyzed total RNA obtained from these cells using reverse transcription polymerase chain reaction (RT-PCR) [166], Piccin and Morshead evaluated cells with active Wnt signaling in brain slices acquired from transgenic mice producing the Wnt/β-catenin-reporter, thus visualizing the canonical Wnt signaling activity [151]. In contrast to both these results, Western blotting showed decreased levels of the β-catenin protein in the mouse brain three days after MCAO [163]. A possible explanation for such discrepancy might be the fact that the whole ischemic hemisphere was used by Yu and colleagues. Nevertheless, the majority of studies agreed on the importance of Wnt signaling in neurogenesis after ischemic injury, and described tissue-detrimental effects of Wnt pathway inhibition on the nervous tissue. Since the behavior of the damaged tissue seems to be very complex, to predict reactions of various cell types on the Wnt stimulus might be quite complicated.

Although NSCs benefit from the activation of the Wnt pathway, the situation is more intricate when it comes to astrocytes. It has been suggested that canonical Wnt signaling is increased in this cell type after ischemic injury. However, its role was linked to post-ischemic seizures, in which astrocytes participate. Interestingly, the inhibition of Wnt signaling in these cells after ischemic injury decreased the otherwise elevated susceptibility to seizures, indicating a possibly detrimental role of Wnt signaling in astrocytes [167]. Additionally, astrocytes have been observed to play a neuroprotective part in ischemic injury via the production of Dkk3, an atypical inhibitor of Wnt signaling. The protective effect was probably mediated by vascular endothelial growth factor (VEGF), and led to increased survival of both neurons and astrocytes [168]. Conversely, a potentially beneficial impact of Wnt signaling activation on astrocytes was observed after treatment with Wnt3a. Ischemic mice treated with this Wnt exhibited higher numbers of astrocytes with the protective phenotype, A2 astrocytes, while the number of astrocytes with the toxic phenotype—A1 astrocytes—was lower [169]. Generally, the effect of Wnt signaling on astrocytes after ischemic injury is still not well documented, and it therefore deserves further examination.

Wnt signaling also influences the reaction of microglia to ischemic conditions. Oxygen-glucose deprivation caused a decrease in the Wnt1 expression in microglial cells. Remarkably, treatment with Wnt1 had a beneficial effect on microglial survival, while the inhibition of Wnt1 increased their apoptosis [170]. Additionally, treating ischemic mice with Wnt signaling activator TWS119 unveiled an effect of the pathway on the microglial phenotype. The microglia of untreated ischemic mice were activated and shifted towards the pro-inflammatory phenotype, whereas the microglia of TWS119-treated mice showed the anti-inflammatory phenotype [171]. Similar results were observed after treatment with Wnt3a, which suppressed the expression of the markers of pro-inflammatory microglial phenotype and potentiated the anti-inflammatory microglial phenotype after ischemic injury. Moreover, the treatment with Wnt3a led to an overall decrease in microglia activation after stroke [169]. Another ligand from the Wnt family, Wnt5a, was produced by astrocytes, and was able to elicit a pro-inflammatory microglial response. This mechanism appeared to act via the Wnt/Ca^2+^ pathway, independently of β-catenin. It is evident that these results provided useful insights into the pro-inflammatory activation of microglia; however, the relevance of the Wnt pathway–microglia crosstalk in the injured brain has not been investigated [172].

The beneficial effects of Wnt signaling were also observed in the cells of oligodendroglial lineage. Myelin repair in the ischemic penumbra was increased after treadmill exercise, and this effect was abolished after the treatment with Wnt signaling inhibitor XAV939, suggesting a possible role of active Wnt signaling in NG2 glia—cells known for their role in remyelination [173]. The differentiation of these cells to myelinating mature oligodendrocytes was also enhanced by the trophic factor tissue inhibitor of metalloproteinases 1 (TIMP1). The protein acted by increasing β-catenin signaling via protein kinase B (Akt) activity. Interestingly, the Wnt7a ligand counteracted the effects of TIMP1 on NG2 glia differentiation, suggesting the convergence of these two pathways [174]. Apart from their role in remyelination, NG2 glia were also observed to affect the BBB function. The transplantation of these cells promoted neurological recovery, decreased the infarct volume and brain edema after ischemic injury, and sustained the integrity of the BBB via activating the canonical Wnt signaling pathway [175].

## 7. The Potential of Wnt Pathway Modulation in the Ischemic Stroke Therapy

In an effort to find a new, more effective treatment for ischemic stroke that would improve the patients’ long-term prognosis and reduce the adverse health consequences, many experimental approaches “came into play”. Some of these approaches, such as treatment with allogeneic mesenchymal bone marrow cells or hyperbaric oxygen therapy, are already being tested in clinical trials (for more information on ongoing clinical trials, please visit the database of privately and publicly funded clinical studies [176]).

In addition to the pharmacological approach, which supports thrombolysis in order to prevent oxygen and nutritional deprivation, the cell-based therapy is a frequently explored possibility. As the neurogenic capacity of NSCs has already been demonstrated beyond doubt, many studies tested the transplantation of embryonic NSCs into the injured brain area. Importantly, several studies, performed not only on rodents but also on experimental pigs and monkeys, have shown the ability of transplanted NSCs to differentiate to long-surviving neurons. Another option is the transplantation of mesenchymal stem cells (MSCs), usually derived from the bone marrow. The disadvantage of these approaches lies in the induction of an immune response in the recipient. A possible solution to this obstacle seemed to be the use of NSCs derived from iPSCs. However, the generation of iPSCs is time-consuming, and their usage is associated with a risk of cancer; both phenomena greatly complicate this therapeutic approach (reviewed in Reference [177]). Since the Wnt signaling pathway promotes NS/PC proliferation and neurogenesis, the molecular support for the patient’s own neurogenesis through Wnt modulatory agents immediately after ischemic stroke comes into consideration as a promising therapy. In fact, some previously proposed methods of stroke treatment might activate Wnt/β-catenin signaling. For example, the neuroprotective function of the melanocortin MC_4_ receptor agonists in neurodegenerative disorders, including ischemic stroke (reviewed in Reference [178]), was linked to the upregulated Wnt and Shh signaling [179]. The cross-talk between these two signaling pathways occurs at multiple subcellular levels. Thus, a deeper understanding of their interactions could be utilized in the search for new chemical substances showing therapeutic potential. A similar positive effect of Wnt/β-catenin signaling on the reduction of neurological defects was demonstrated after administration of the non-steroid anti-inflammatory agent sulindac in adult rats after MCAO [180], or with glucagon-like peptide 1 (GLP1) analog liraglutide [181]. The latter is currently used in the treatment of type 2 *diabetes mellitus* and obesity. Additionally, hyperbaric oxygen therapy, which is used in decompression sickness or long-lasting wound treatment, also showed neuroprotective effects after stroke. An increase in neurogenesis and neurological functions, observed after treatment with hyperbaric oxygen, was linked to the upregulated HIF1α levels and, consequently, to increased β-catenin stability [182]. Moreover, the hyperbaric oxygen therapy promoted the differentiation of NSCs not only to neurons, but also to oligodendrocytes, and this effect was attenuated by Wnt signaling inhibitors [183]. Interestingly, *Alpina officinarum*-derived flavonoid galangin improved neuronal survival in mice after MCAO. The neuroprotective effect on the neurovascular unit was mediated by the HIF1α/VEGF and Wnt/β-catenin pathways [184]. Similarly, other plant derivatives, such as phenolic glycoside gastrodin and iridoid glycoside cornin, inhibited apoptosis and induced Wnt3a expression [185], and increased angiogenesis and Wnt5a expression [186], respectively, in the ischemic region of the mouse brain. Strikingly, electrical stimulation or electro-acupuncture, used in traditional Chinese medicine for the prevention and treatment of stroke, also seems to stimulate the proliferation of NSCs via the canonical Wnt pathway. Surprisingly, this therapy reduced the cerebral infarct volume and alleviated subsequent neurological deficits [187]. The effect of the canonical Wnt signaling pathway on the CNS is mainly associated with cell proliferation, migration and differentiation, as well as with the regulation of inflammation after acute injury (reviewed in Reference [188]). It is therefore a matter of discussion to what extent the amelioration of the pathological changes provoked by ischemic stroke is directly related to the activation of the Wnt pathway. Alternatively, increased Wnt signaling might be “a side effect” of the anti-inflammatory and pro-proliferative processes triggered in the damaged tissue.

Interestingly, several Wnt inhibitors, including Dkk proteins or miRNAs, appear to be produced in the adult brain after stroke, and contribute to neuronal degradation [145,161]. The suppression of these negative Wnt pathway regulators could “unlock” or “restart” the neurogenic potential of the adult brain, and thus contribute to the regeneration of the ischemic brain regions. Estrogen hormonal therapy suppressed Dkk1 expression in the tissue, and thus promoted the Wnt pathway’s activity when administered shortly after ischemic injury. Such therapy also has some disadvantages; namely, the endogenous estrogen level may play an important role. It is not uncommon for postmenopausal women to fall into a state of estrogen deficiency, and thus the brain’s sensitivity to estrogen is lost and the effect of hormonal therapy is markedly reduced (reviewed in Reference [189]). The volume of the ischemic lesion in mice was reduced by the administration of the miRNA-148b inhibitor, leading to improved neurological functions [161]. Furthermore, recent research has demonstrated that extrinsic Wnt/β-catenin pathway stimulation by synthetic ligands or chemical compounds performed immediately after ischemic stroke was capable of reducing the detrimental health consequences of the insult. The administration of Wnt3a directly to the neurogenic region of the brain promoted the proliferation of NS/PCs, which subsequently significantly reduced the extent of damage and improved the functional recovery of mice after MCAO [153]. A similar outcome was noted using intranasal administration of the Wnt3a ligand, whereas this effect was reversed by the co-administration of Wnt pathway-specific inhibitors, such as Dkk1 or tankyrase inhibitor XAV-939 [152]. It should be emphasized that these studies are still in their infancy, and most of the data were obtained using experimental rodent models. Importantly, the stimulation of the Wnt pathway could accompany the existing stroke treatment, and thus improve the patient’s medical outcome. One recent study showed that administration of the Wnt pathway stimulator GSK3β inhibitor TWS119 reduced the risk of hemorrhagic transformation, a serious health complication upon treatment of ischemic patients with thrombolytic agent tPA [190]. Consistently, a number of studies have revealed the significant role of the Wnt/β-catenin pathway in suppressing inflammation, inducing immune tolerance in dendritic cells, and also improving the BBB integrity (reviewed in Reference [191]). We conclude that the stimulation of the Wnt pathway after ischemic stroke could not only promote neurogenesis in the ischemic brain, but might also support other processes necessary for successful patient recovery.

## 8. Future Perspectives

Aberrant Wnt signaling underlies many types of human diseases, including cancer, and therefore a great effort has been made to identify the negative regulators of the signaling. On the other hand, a number of synthetic activators of the Wnt pathway were also (co)discovered (reviewed in Reference [192]). Since these activators might improve the recovery of patients after ischemic stroke, it is quite possible that the targeting of the Wnt pathway after ischemic stroke will enter clinics in the near future. However, one must always keep in mind that the Wnt signaling pathway’s activity is not only essential for stem cell maintenance, but the pathway also promotes cell division, leading in some (extreme) situations to cellular transformation (reviewed in Reference [193]). This then raises the following questions: Is it safe to (hyper)stimulate the Wnt pathway? Does the risk of such treatment outweigh the benefits to the patient who suffered from ischemic stroke? We anticipate that, to answer these questions, a good understanding of the signaling pathways operating in the brain cells and tissue is essential.

Although a large amount of experimental data has already been obtained, it is clear that further experiments will be needed to be able to fine-tune the signaling level of the Wnt signaling pathway. The results of these experiments should maximize the regeneration of the damaged brain, without any adverse effects on the patient’s body.

## Abbreviations

Akt	protein kinase B
AP1	activator protein 1
APC	adenomatous polyposis coli
ATP	adenosine triphosphate
ATP6AP2	ATPase H^+^ transporting accessory protein 2
AXIN	axis inhibition
β-CAT	β-catenin
β-TrCP	β-transducin repeats-containing protein
BBB	blood–brain barrier
Bcl2	B-cell lymphoma 2
BDNF	brain-derived neurotrophic factor
BMP2/4	bone morphogenetic proteins 2 and 4
C-JUN	transcription factor C-JUN
Ca^2+^	calcium
CaMKII	Ca^2+^/calmodulin-dependent protein kinase II
CC	corpus callosum
CDC42	GTPase CDC42
CELSR1	cadherin epidermal growth factor laminin G seven-pass G-type receptor 1
CK1	casein kinase 1
Cl^−^	chloride
CNS	central nervous system
CSF	cerebrospinal fluid
CTX	cortex
DAAM1	DVL-associated activator of morphogenesis 1
DAG	diacylglycerol
DAPI	4′,6-diamidino-2-phenylindole
DCX	doublecortin
DG	dentate gyrus
Disc1	disrupted in schizophrenia 1
DKK1/3	dickkopf 1/3
DVL	dishevelled
ER	endoplasmic reticulum
FACS	fluorescence-activated cell sorting
FCI	focal cerebral ischemia
FZD	frizzled
GCI	global cerebral ischemia
GCL	granule cell layer
GFAP	glial fibrillary acidic protein
GLI1,2,3	glioma-associated oncogene 1,2,3
GLP1	glucagon-like peptide 1
GSK3β	glycogen synthase kinase 3β
HIF1α	hypoxia-inducible factor 1α
HIP	hippocampus
Hipk1	homeodomain interacting protein kinase 1
IP3	inositol trisphosphate
iPSCs	induced pluripotent stem cells
JNK	c-Jun N-terminal kinase
LGR5	leucine-rich repeat-containing G protein-coupled receptor 5
LiCl	lithium chloride
LINE1	long interspersed nuclear elements 1
LRP5/6	low-density lipoprotein receptor-related protein 5/6
LVs	lateral ventricles
MAPK	mitogen-activated protein kinase
MBP	myelin basic protein
MCAO	middle cerebral artery occlusion
Meg3	maternally expressed gene 3
miRNA	microRNA
MSCs	mesenchymal stem cells
Na^+^	sodium
NeuroD1	neurogenic differentiation 1
NFAT	nuclear factor of activated T-cells
NG2	neuron-glial antigen 2
NLK	nemo-like kinase
NPCs	neural progenitor cells
NS/PCs	neural stem/progenitor cells
NSCs	neural stem cells
OPCs	oligodendrocyte precursor cells
P	phosphorylation
PCNA	proliferating cell nuclear antigen
PCP	planar cell polarity
PDGFRα	platelet-derived growth factor receptor α
PIP2	phosphatidylinositol 4,5-bisphosphate
PKC	protein kinase C
PLC	phospholipase C
Prox1	prospero-related homeodomain transcription factor 1
RAC1	Rac family small GTPase 1
RhoA	Ras homolog family member A
ROCK	Rho-associated kinase
ROR	receptor tyrosine kinase ROR
ROS	reactive oxygen species
RT-PCR	reverse transcription polymerase chain reaction
RYK	receptor tyrosine kinase RYK
sFRPs	secreted FZD-related proteins
SGZ	subgranular zone
Shh	sonic hedgehog
siRNA	small interfering RNA
Sox2	SRY-box transcription factor 2
STR	striatum
SVZ	subventricular zone
TCF/LEF	T-cell factor/lymphoid enhancer-binding factor
TIMP1	tissue inhibitor of metalloproteinases 1
TLE	transducin-like enhancer of split
tPA	tissue plasminogen activator
Ub	ubiquitination
VEGF	vascular endothelial growth factor
VW	ventricular wall
WIF	Wnt inhibitory factor
Wip1	wild-type p53-induced phosphatase 1
WLS	Wntless
